# Advance hybrid medical watermarking algorithm using speeded up robust features and discrete cosine transform

**DOI:** 10.1371/journal.pone.0232902

**Published:** 2020-06-04

**Authors:** Saqib Ali Nawaz, Jingbing Li, Uzair Aslam Bhatti, Anum Mehmood, Muhammad Usman Shoukat, Mughair Aslam Bhatti

**Affiliations:** 1 College of Information and Communication Engineering, Hainan University, Haikou, China; 2 State Key Laboratory of Marine Resource Utilization in the South China Sea, Hainan University, Haikou, China; 3 School of Geography, Nanjing Normal University, Nanjing, China; 4 College of Biology, Institute of Tropical Agriculture and Forestry, Hainan University, Haikou, Hainan, China; 5 Laboratory of Biotechnology and Molecular Pharmacology, Hainan Key Laboratory of Sustainable Utilization of Tropical Bioresources, Hainan University, Haikou, Hainan, China; 6 School of Automation and Information Sichuan University of Science and Engineering, Yibin, China; 7 Hamdard University, Karachi, Pakistan; National Textile University, PAKISTAN

## Abstract

In the continuous development of computer network technology, multimedia technology and information technology, digitization has become the main means of displaying information, thus facilitating the storage, copying and dissemination of digital multimedia information. In this context, there are no restrictions on arbitrary editing, copying, modification, and dissemination of digital images, music, etc., which leads to various social problems such as information security, copyright disputes, and piracy. With the advancement of networks and multimedia, digital watermarking technology has received worldwide attention as an effective method of copyright protection. Improving the anti-geometric attack ability of digital watermarking algorithms using image feature-based algorithms have received extensive attention. This paper proposes a novel robust watermarking algorithm based on SURF-DCT perceptual hashing (Speeded Up Robust Features and Discrete Cosine Transform), namely blind watermarking. The algorithm firstly uses the affine transformation with a feature matrix and chaotic encryption technology to preprocess the watermark image, enhance the confidentiality of the watermark, and perform block and DCT coefficients extraction on the carrier image, and then uses the positive and negative quantization rules to modify the DCT coefficients. The embedding of the watermark is completed, and the blind extraction of the watermark realized. Experiments show that the algorithm has good invisibility and strong robustness against conventional and geometric attacks and can effectively protect the security of images with NC value more than 90%.

## 1. Introduction

In recent years, with the development of network communication and multimedia technology, the copyright protection and content authentication of digital products have received more and more attention. Digital watermarking as an effective means to protect the copyright and integrity of digital products, how to ensure the protection of the copyright of digital products It is crucial that the information is robust and resistant to detection. Digital watermarking is an effective way to realize the copyright of digital works by embedding secret information (watermarks) in the original data to confirm the ownership of the work. [1].

The research of digital watermarking technology has gradually become a hot issue in the field of image processing. Through this technology, the purposes of copyright protection, tampering positioning and integrity protection can be achieved. The watermark system should meet the requirements of invisibility and robustness at the same time, but these two are contradictory [2]. In recent years, many anti-geometric attack algorithms have emerged, making anti-geometric attack watermarking algorithms a hot topic of research. Such algorithms generally use the invariance of feature points, or the invariance of moments to correct the image under attack, and then extract the watermark. In order to effectively improve the ability of the image watermarking algorithm to resist geometric attacks, the second-generation image watermarking technology based on image feature points has received extensive attention [3].

**Fig 1:**
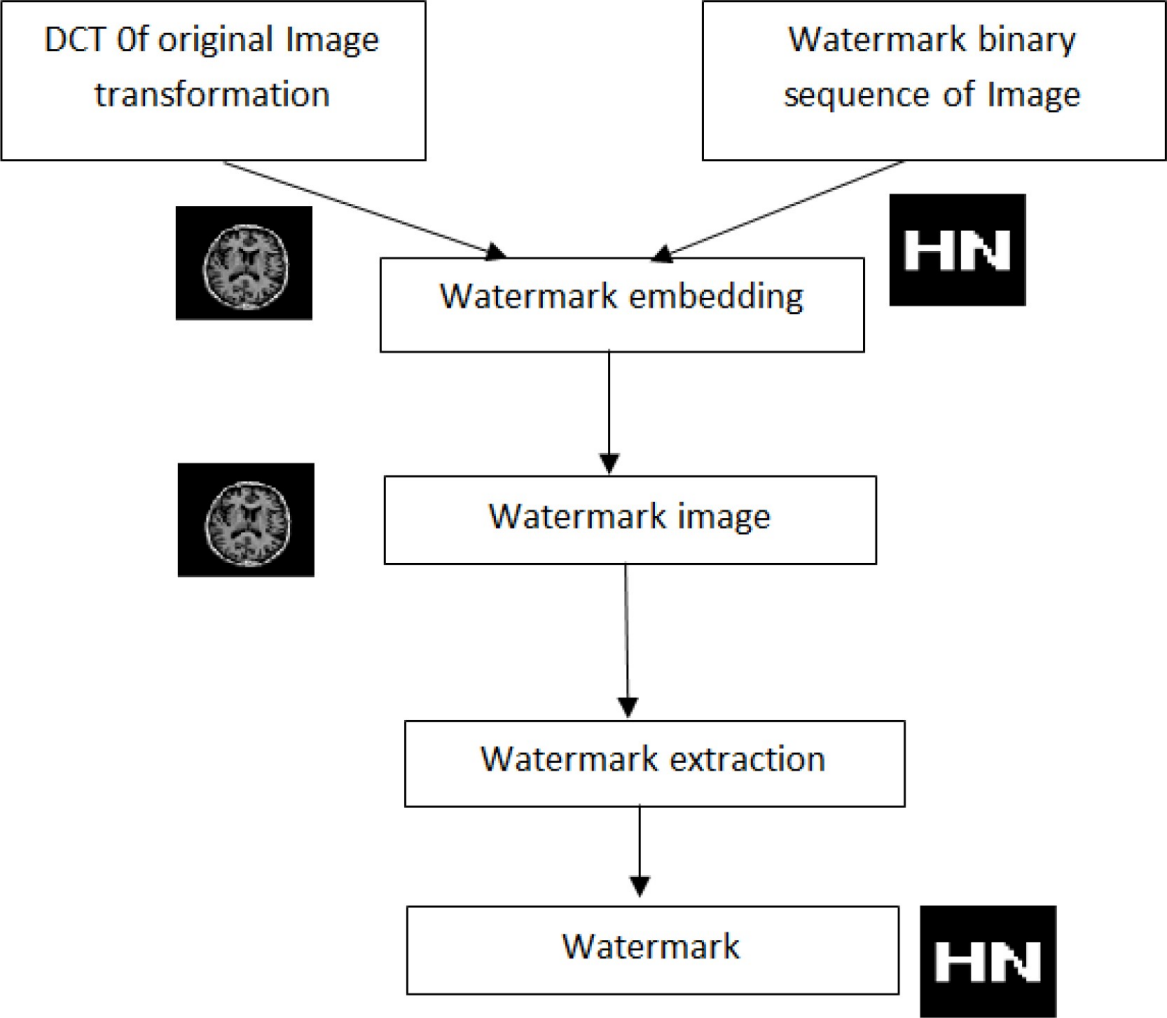
Watermarking steps using a DCT coefficient.

Digital watermarking is a new modern technology, which refers to the important content of information hiding technology in the research process [4]. Digital watermark can effectively protect the actual copyright, and it has become a key content in the research of multimedia information security [5]. Digital watermark can use the corresponding algorithm to integrate secret information into the protected digital multimedia, that is, watermark, which represents the behavior of copyright ownership and tracking infringement [6]. By analyzing and designing the spatial domain and transform domain of the digital watermarking algorithm, it can effectively determine the block DCT digital image watermarking algorithm, such as generating watermark, embedding watermark, extraction and detection, etc. In the specific use process, the watermark image is generated by DCT transform, attacking the watermark image and verifying the robustness. DCT watermarking steps are shown below in Fig 1.

The existing DCT-based watermarking algorithm changes a DCT coefficient with a particular rule for each embedded 1-bit information. Thus, the DCT coefficient that changes in the entire image is at least equal to the binary sequence number of the watermark [7]; however, this is in terms of improved correlation values of the image, which affects the invisibility of the watermark to some extent.

The main contribution of this research is as follows:

- Secured medical image watermarking algorithm which is strong against the geometric attacks as well as conventional attacks.
- Blind watermarking which prevents the security of watermark after embedding and also help in restoring the image.
- Higher NC value shows the robustness of our watermarking algorithm and satisfy the requirement of the invisible watermarking.

The remainder of the paper is divided as follows: In the next section, all relevant developed methods of digital image watermarking were compared with the proposed algorithm; then, basic concepts regarding SURF and DCT discussed. Subsequently, a proposed algorithm and experiment were performed, followed by a conclusion and future research directions.

## 2. Related work

SURF is a powerful image detector and descriptor, first proposed by Herbert Bay [8][9], and partly inspired by the Scale-Invariant Feature Transform (SIFT). Another work [10] used the SIFT algorithm to extract the feature points of the carrier image and estimated and corrected the affine parameters of the attacked carrier image. Although the extracted watermark has good robustness, the SIFT algorithm requires a large amount of calculation and runs low efficiency. Adding the encrypted signature [11] in the center location of the image is another technique of embedding watermarking in blocks of 8*8 DCT segments, which decrease computational complexity with increase in security. Another approach [12] is proposed finding the significant region of watermark and embedding the watermark after using the pseudorandom sequence generation for improved security which gives a compound image. A detailed techniques of watermarking is proposed in [13] which gives the overview about the ways of watermarking in images.

A semi-blind video watermarking algorithm [8] using block classification is effective with SURF and provides strong robustness for the security of images and copes with most attacks. Image tampering [9] used the Speed-Up Robust Features algorithm to extract stable feature points and feature point descriptors in low frequency subbands of images and used feature point matching to estimate and correct geometric attacks on watermarked images parameter. However, the coefficients of the ROI and ROB regions cannot be separately embedded in the watermark, and the quality of the restored carrier image after extracting the watermark is not high. Mohanty et al. [14] proposed the approach of merging the visible and invisible watermarking technique which is effective in detecting the tampering in images. Dividing the image into different block and then embedding that watermark in those blocks is useful technique and we proposed algorithm based on similar approach by dividing the image in different blocks of low frequency and high frequency.

**Fig 2:**
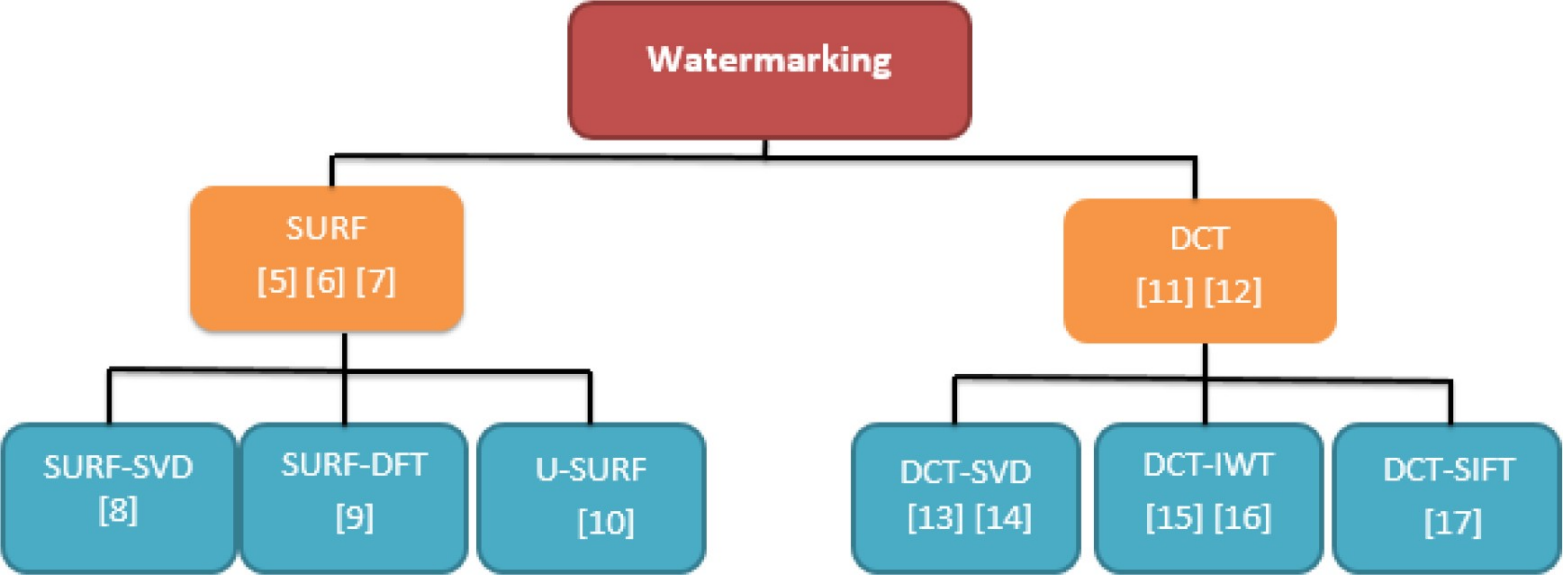
DCT watermarking transformation workflow.

Another SURF approach with KNN Dimensional (KD Tree) [15] is effective in identifying the duplicated regions of the multidimensional data. SURF is firstly used for extracting out the features of the image. The second step is to link the keypoint matching, trailed by a typical mode check step channel pair. The KD tree is used to search for nearest neighbors in an image block. However, that approach uses Euclidean distance for measuring the nearest neighbors, which may be time-consuming if the image pixels ratio and color samples are high. Wang Wei [16] proposed a new method for finding feature regions for watermark insertion, then performing Singular Value Decomposition (SVD) on each region and embedding a watermark. Finally, the SURF descriptor is extracted from the watermark image and saved for future attack type estimation and attack image correction, ensuring that the watermark embedding position is stable and the watermark can be accurately extracted. However, slow and computationally excessive consumption of memory by SVD provides poor results [17][18]. Cedillo-Hernandez et al. [19] proposed a new method by using the discrete Fourier transform (DFT) whereby watermarking is done by embedding it in the middle frequency band amplitude of the image and then using SURF for selecting object regions as features which helps in restoring the watermark even if the image is distorted. In order to identify the selected object area after geometric distortion, the SURF features are pre-estimated and stored for use during the detection and embedding process. In the detection phase, the SURF features of the distorted image are estimated and matched to the stored features. Based on the matching result, the SURF feature is used to calculate the affine transformation parameters and restore the object area. Despite being a good embedding technique, it is time-consuming, which affects performance. Subsampling [20][21][22] of the watermark region is effective using DCT. It is also possible to increase the security of the image by inserting a watermark in the sub-image. A discrete cosine change area (DCT domain) watermarking method like spread range can give powerful copyright security to advanced static images [23][24]. The summed-up Gaussian circulation measurably models the DCT coefficients of the first image and shows how the subsequent indicator structure results in a critical improvement in the presentation of the related beneficiary, which has been broadly considered in the writing and uses Gaussian noise presumptions. Sverdlov et al. reason another vigorous mixture watermarking plan dependent on DCT and SVD [25]. The block watermark is embedded in the low frequency sub-band coefficients of the region of interest. The algorithm fails to recover the data in the region of interest after extracting the watermark, which is an irreversible watermark. The singular value represents the essential characteristics of an image. After the watermark is embedded, the singular value changes little when the image is subjected to a simple attack. Fig 2 shows the hierarchical progress of different techniques of the watermark. Using DCT [26] blocks of 8*8 is another useful approach using Geometric Algebra (GA) technique and incorporating it with extreme adjustment pixels (ADP). This approach using Quaternions which is the sub algebra of the GA and effective in processing vector based image processing. Approach use the bits embedding in the different transformation components of vectors. That processing of watermark requires efficient GA processing and complexity is high.

Multiple watermarking refers to the technique of embedding multiple watermarks in various ways in the same carrier of the image but at different frequencies. Similar multiple watermarking algorithm technique is used by Roy [27], he embeds the watermark in different color bands using DCT and repetition code. Embedding the watermark in different color bands is effective in creating the robust and secure watermark but it increases the computational time and complexity. Our proposed algorithm in this paper is also effective for multiple watermarking. The proposed algorithm is based on using DCT with SURF, which helps in embedding the watermark at a lower frequency. The main benefit of this type of embedding is that it provides blind extraction as well as zero watermarking. It embeds multiple watermark identifiers into the carrier in various ways, which improves the robustness and security of the watermark from different aspects.

### 3. The fundamental theory

### A.  Discrete Cosine Transform (DCT)

Discrete cosine transforms, especially its second type, are often used for signal processing and image processing for lossy data compression of signals and images, including still and moving images. This is because the discrete cosine transform has a strong "energy concentration" characteristic: most of the natural signals (including sound and image) are concentrated in the low-frequency part of the discrete cosine transform [28]. Discrete Cosine Transform is an orthogonal transform with strong de-correlation capability. The image after DCT transform has strong energy concentration characteristics, mainly concentrated in the digital communication (DC) and low frequency parts after Discrete Cosine Transform, in the frequency domain The low-frequency amplitude of the upper left corner of the matrix is large and the high-frequency amplitude of the lower right corner is small. The low-frequency part represents the contour and grayscale distribution characteristics of the image, and the high-frequency part represents the details and edge information of the image shape. Considering the case of the grayscale image f(x, y), 0 ≤ x, y ≤ N-1, the forward two-dimensional DCT can be expressed as [29].

$$F\left(u,v\right)=\alpha \left(u\right)\alpha \left(v\right)\overset{N-1}{\underset{x=0}{\sum }}\overset{N-1}{\underset{y=0}{\sum }}f\left(x,y\right)\cos\left[\frac{\left(2X+1\right)\pi }{2N}u\right]\cos\left[\frac{\left(2y+1\right)\pi }{2N}v\right]$$ (1)

Where u, v = 0, 1, 2,…, N-1. The 2D DCT is symmetrical and an orthogonal transform. α(u), α(v), and inverse 2D DCT are defined in (2) and (3).

α(u)={1Nu=02/Nu≠0α(v)={1Nv=02/Nv≠0 (2)

$$f\left(x,y\right)=\overset{N-1}{\underset{x=0}{\sum }}\overset{N-1}{\underset{y=0}{\sum }}\alpha \left(u\right)\alpha \left(v\right)f\left(u,v\right)\cos\left[\frac{\left(2X+1\right)\pi }{2N}u\right]\cos\left[\frac{\left(2y+1\right)\pi }{2N}v\right]$$ (3)

**Fig 3:**
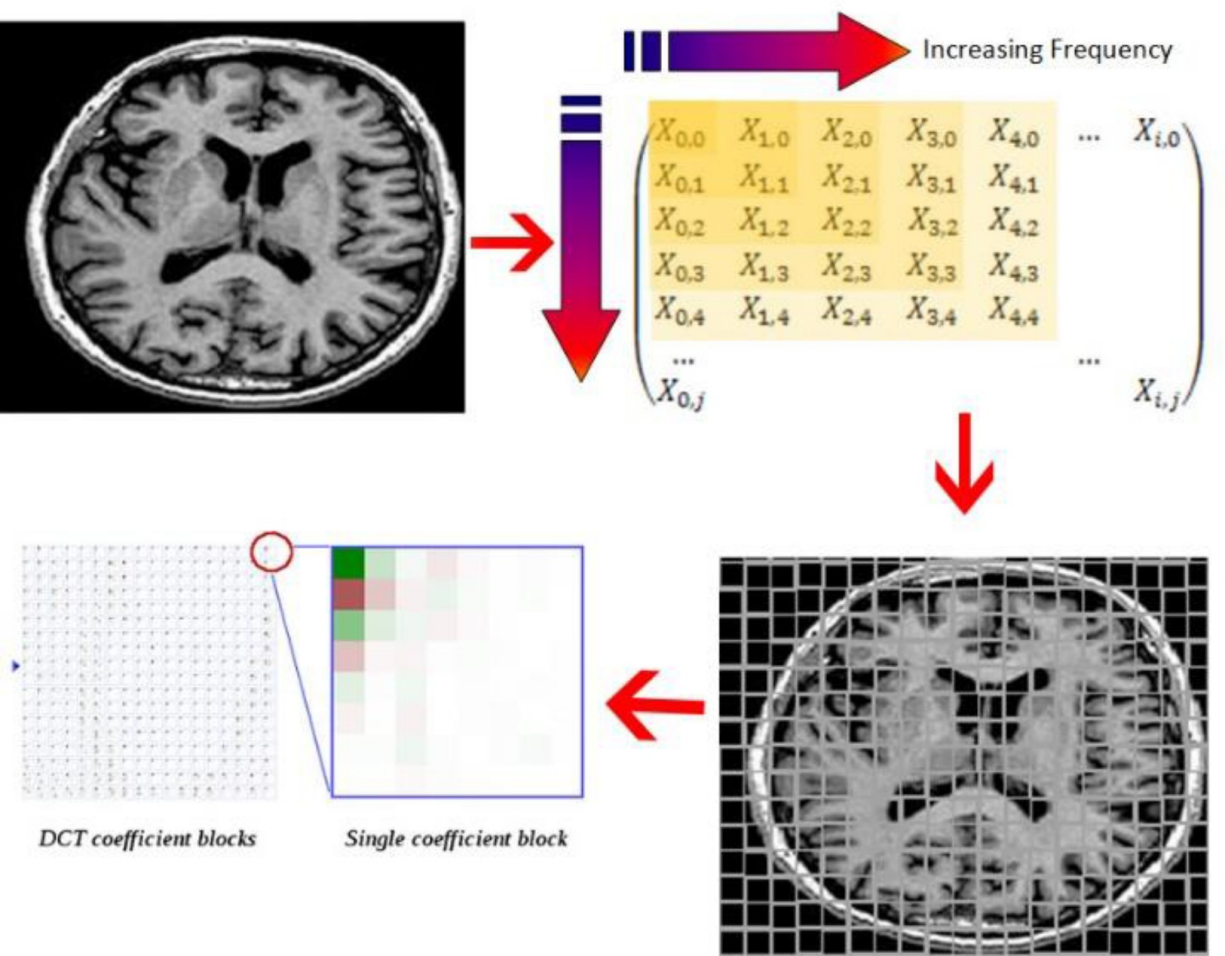
DCT coefficient for matrix factorization.

The DCT helps to divide the image into different frequency bands, such as low frequency (LF), intermediate frequency (MF), and high frequency (HF) of different importance (relative to the visual quality of the image with its feature matrix), as shown in Fig 3. Using those bands with SURF individually will help in finding the relevant features of each frequency [30], discussed in the next section.

Watermark embedding in the DCT area can be communicated as:

$$F_{s}^{/}=F_{s}Q$$ (4)

Where *Fs* and *Fs'* are matrices selected for embedded DCT coefficients and corresponding modified DCT coefficients, and Q is a set of weighting factors provided by the watermark signal.

### B. Speedup Robust Feature (SURF)

After selection of particular frequencies with their vector matrices, we use SURF to find the area of interest to embed a watermark.

#### 1) Feature selection of image

When the SURF algorithm extracts feature points, an integral image is first introduced to simplify the calculation of the image area. The integral image is defined as the value of any point P(i,j) in the integral image, and n(i,j) is the sum of the gray values of the diagonal regions corresponding to the respective upper left corner of the original image. The integral image formula is:

$$n(i,\;j)=\sum_{i^{\prime }<i,j^{\prime }<j}P\left(i^{\prime },j^{\prime }\right)$$ (5)

**Fig 4:**
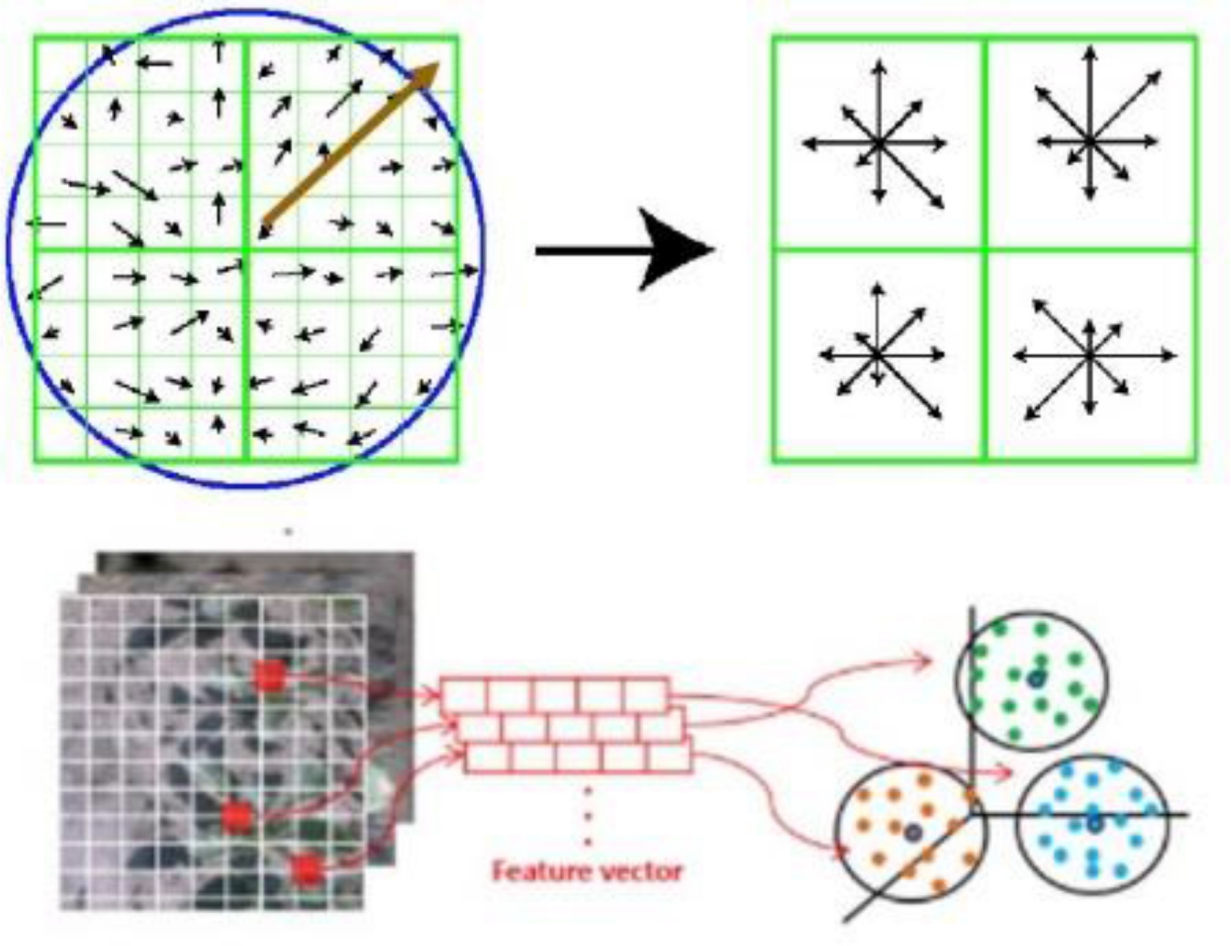
SURF feature extraction and clustering.

Then, in the original image, the sum of the gradations in the rectangular region can be converted into addition and subtraction of the integrated image, which reduces the computational complexity. SURF makes it is easy to categorize the feature according to the nature of frequencies by using DCT, as shown in Fig 4.

#### 2) Hessian matrix detection feature points

The SURF algorithm uses the local maximum of the Hessian matrix determinant to locate the feature points. Assuming that X(x, y) is a point in image I, the Hessian matrix is defined at scale σ as:

$$H\left(X,\sigma \right)=\left[\begin{array}{ll} L_{\mathrm{xx}}(X,\sigma ) & L_{\mathrm{xy}}(X,\sigma )\\ L_{\mathrm{xy}}(X,\sigma ) & L_{\mathrm{yy}}(X,\sigma ) \end{array}\right]$$ (6)

If the discriminant of the Hessian matrix has a local maximum, the current point is brighter or darker than the surrounding points, and it can be judged as a candidate extreme point. In order to improve the operation speed, SURF uses a box filter to approximate the Gaussian filter. When locating the extreme point, the Hessian discriminant of each pixel needs to be calculated. If it is a positive number, the pixel is a local extreme point, otherwise it is not. The obtained extreme point is used as a candidate feature point.

$$Det(H)=D_{\mathrm{xx}}D_{\mathrm{yy}}-(\omega D_{\mathrm{xy}}{)}^{2}$$ (7)

#### 3) Chaotic Mapping

Chaos is a complex non-dynamic equation. When the parameters are within a controllable range, it will exhibit a state of aperiodic behavior that is sensitive to the initial conditions. This characteristic is well received in the field of image encryption. Chaotic Map is relatively simple to implement and is the most widely used and most typical chaotic system. The mathematical expression is as follows:

f(x)=(k×x)modN+1 (8)

In the formula, it is called the Logistic parameter, which is a system variable, and k is the number of iterations. The mapping of Eq (8) is a piecewise linear function with significant regularity. Its regularity leads to a relatively poorly confusing effect, and an attacker can attack the watermark through the law of this mapping. Therefore, the algorithm uses a new chaotic map as follows:

$$\{\begin{array}{l} x_{i}=1-a_{i-1}^{2}\\ y_{i}=\cos(\beta \;\mathrm{arc}\;\cos\;x_{i-1}) \end{array}$$ (9)

where: x, y ∈ [–1, 1], α and β are two controllable variables. In this paper, a chaotic sequence is generated iteratively for Eq (3), and the y variable sequence is reserved for the binary watermark image to be chaotically processed.

## 4. Proposed algorithm

The proposed algorithm uses DCT-SURF with perceptual hashing to provide security for the images.

### A. Feature selection

**Fig 5:**
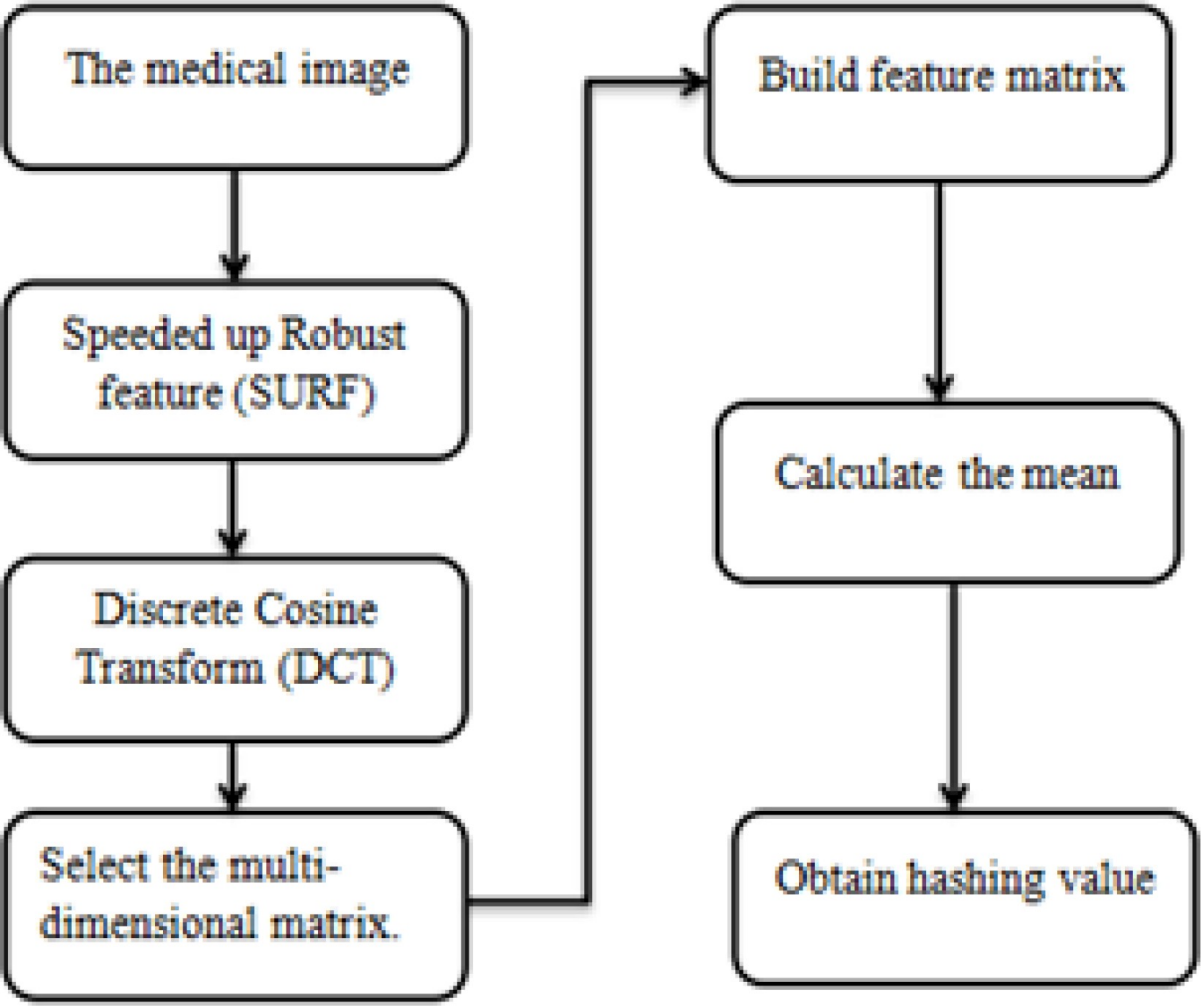
SURF for feature selection.

The perceptual hash algorithm is a general term for a class of algorithms, including aHash (average hash and faster), pHash (perceived hash and high accuracy), and dHash (dual property). In this hashing, the hash value is not calculated in a very strict manner, but rather computes hash values more relatively, therefore provides similarity between the images based on features. At this time, comparing the similarity of the two images is to first calculate the hash fingerprint of the two images, that is, the 0 or 1 value, and then calculate the number of different bits (Hamming distance). If the value is 0, it means that the two images are very similar. If the Hamming distance is less than 0.5, it means that it is somewhat different, but it is similar. If the Hamming distance is greater than 0.5, it means it is an entirely different image. The mean hash is a simple way to calculate the feature vector matrix and helps to get the final embedded image. There is a more robust algorithm called pHash, which takes the average value of each feature vector so that a uniform watermark is embedded in all areas of an image. A discrete cosine transforms (DCT) is used to obtain the low-frequency components of the image. At the same time, the binarization rules are as follows:

$$h\left(n\right)=\{\begin{array}{c} 1\;,if\;s\left(i,j\right)\geq m\\ 0\;,if\;s\left(i,j\right)<m \end{array},\;i\;\epsilon \left[M_{1,}M_{2}\right]j\;\epsilon \left[N_{1,}N_{2}\right]$$ (10)

where S (i, j) speaks to the relating cell esteem in the image lattice. The m is the mean of the sub-modules. Fig 5 demonstrates the calculation stream. The particular advances are as follows:

Step 1. The main relative features were identified using perceptual hashing.

Step 2. DCT was applied for transformation.

Step 3. The transformation selected features.

Step 4. A multidimensional matrix was created from those features.

Step 5. Computing the mean to get the value of the hash function of those features (refer Fig 5).

### B. Preprocessing of the watermark

**Fig 6:**
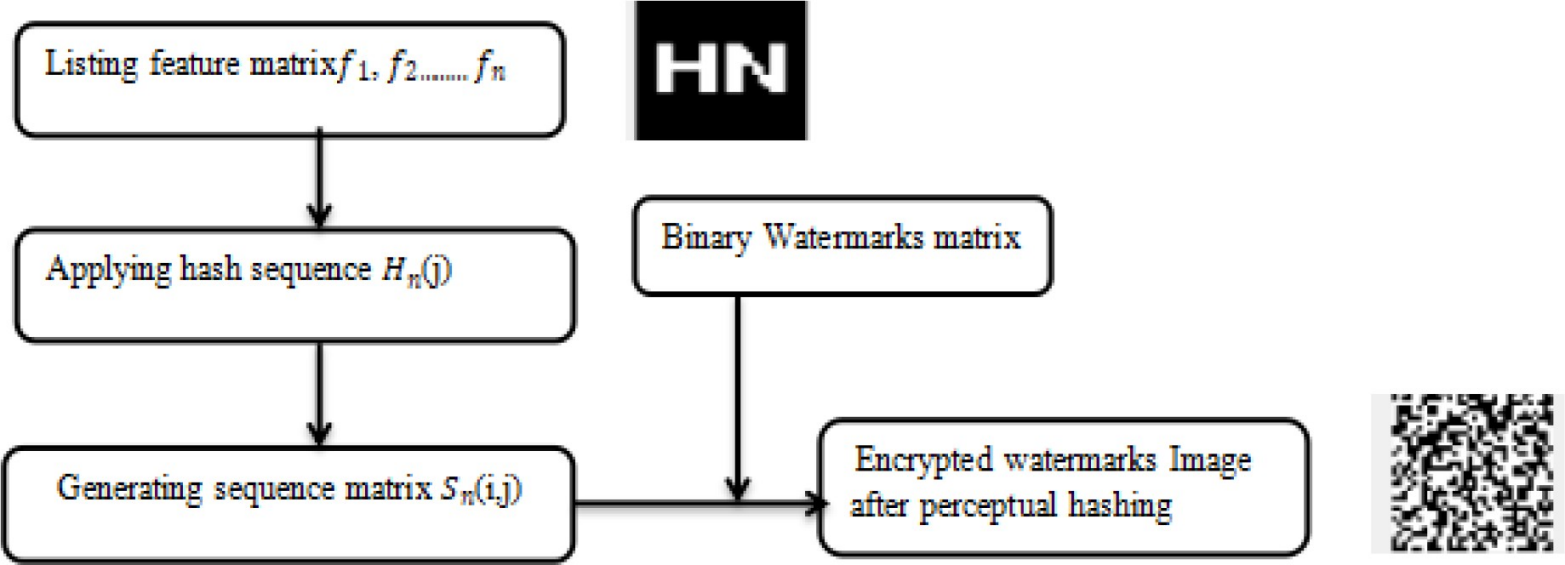
Feature matrix implemented by a hash sequence function.

The watermark is preprocessed to get ready for embedding on the feature’s matrix. Different multidimensional features were analyzed before embedding the watermark. A hashing map is applied to find the collection for storing key-value pairs. These key-value pairs are called Feature Entry. A hash is very similar to an array. The primary difference is that the array directly accesses the data of a specific location through a specific index. The hash table first obtains the corresponding index through the hash function and then locates the corresponding index. storage location. This is a relatively low-level knowledge. The general hash table is stored in an array at the bottom. The biggest advantage of using an array as the underlying data structure is its random-access characteristics. A hashing map matrix is generated by storing those feature entries in the array. Every feature entry is stored in a hash map matrix array for the generation of a matrix, which is a multidimensional array of the feature vector. The watermark preprocessing steps are shown in Fig 6.

Step 1: Feature matrix *f1*,*f2……*,*fx* is created from sequence matrix *Sn(i*, *j)* and hash sequence *Hn(j)* by using the symbolic operations.

Step2: Encrypted watermark *CSn(i*,*j)* is obtained after hash function implementation on matrix *Sn(i*,*j)*.

Watermark embedding:

The watermarking involves the method of repeatedly and redundantly embedding on different places to make it hidden and secure without disturbing the original images.

The following are the watermark embedding steps.

Step 1. Generating a key function pseudo-random sequence of length for creating the watermark.

$$N=\left\{w_{i},\;where\;i=1,2,\ldots N\right\},\;and\;w_{i}\in \left\{0,1\right\}$$ (11)

Step 2. DCT coefficient matrix locating.

Step 3. SURF detection operator used for extracting the image and finding the watermark location, which needs to be filled out by that watermark (i.e., "zero fill").

Step 4. Generating the appropriate length of the disordered arrangement to scramble the first watermarking image.

**Fig 7:**
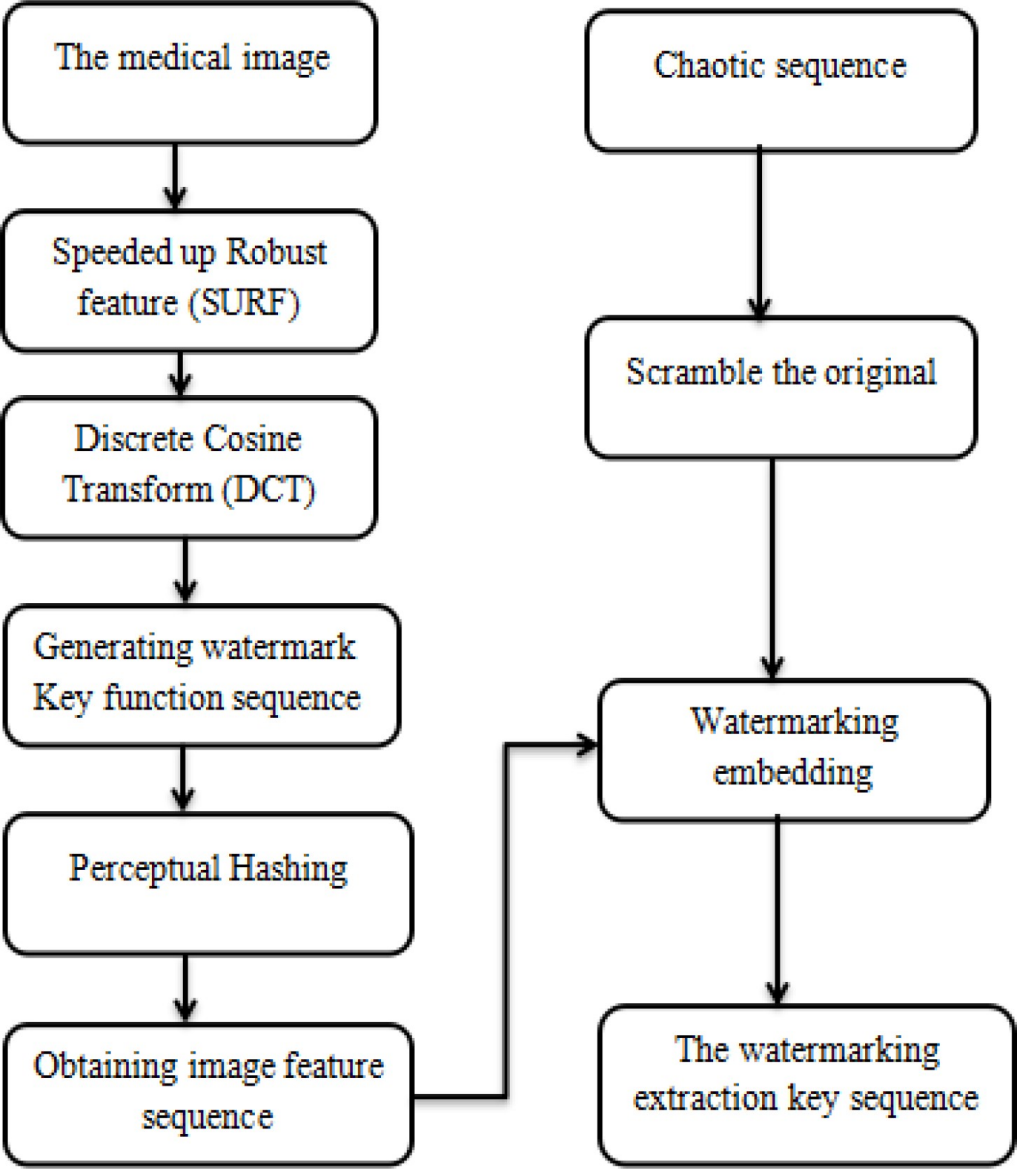
The flow of watermark embedding.

Step 5. Generating the watermarking extraction key arrangement with the sequencing activity, as shown in Fig 7.

### C. Extraction of watermark

Because the addition of the watermark is superimposed on the frequency domain of the original image, we only need to perform reverse transform on the original image and the watermarked image, respectively, and finally, extract the encrypted watermark data through subtraction. The key steps for watermark extraction follow:

Step 1. SURF-DCT could handle the attacked medical image.

Step 2. A watermarked image is first subjected to multiple binary non-overlapping partitioning of sequences, and each block is subjected to multi-dimensional DCT. Then, the position of the watermark-embedded block is found based on the key: 1, 2 … N.

Step 3. Decrypting the extracted watermark sequence from a low-frequency band using key bit by bit reverse hashing and converting the decoded binary data into an image.

**Fig 8:**
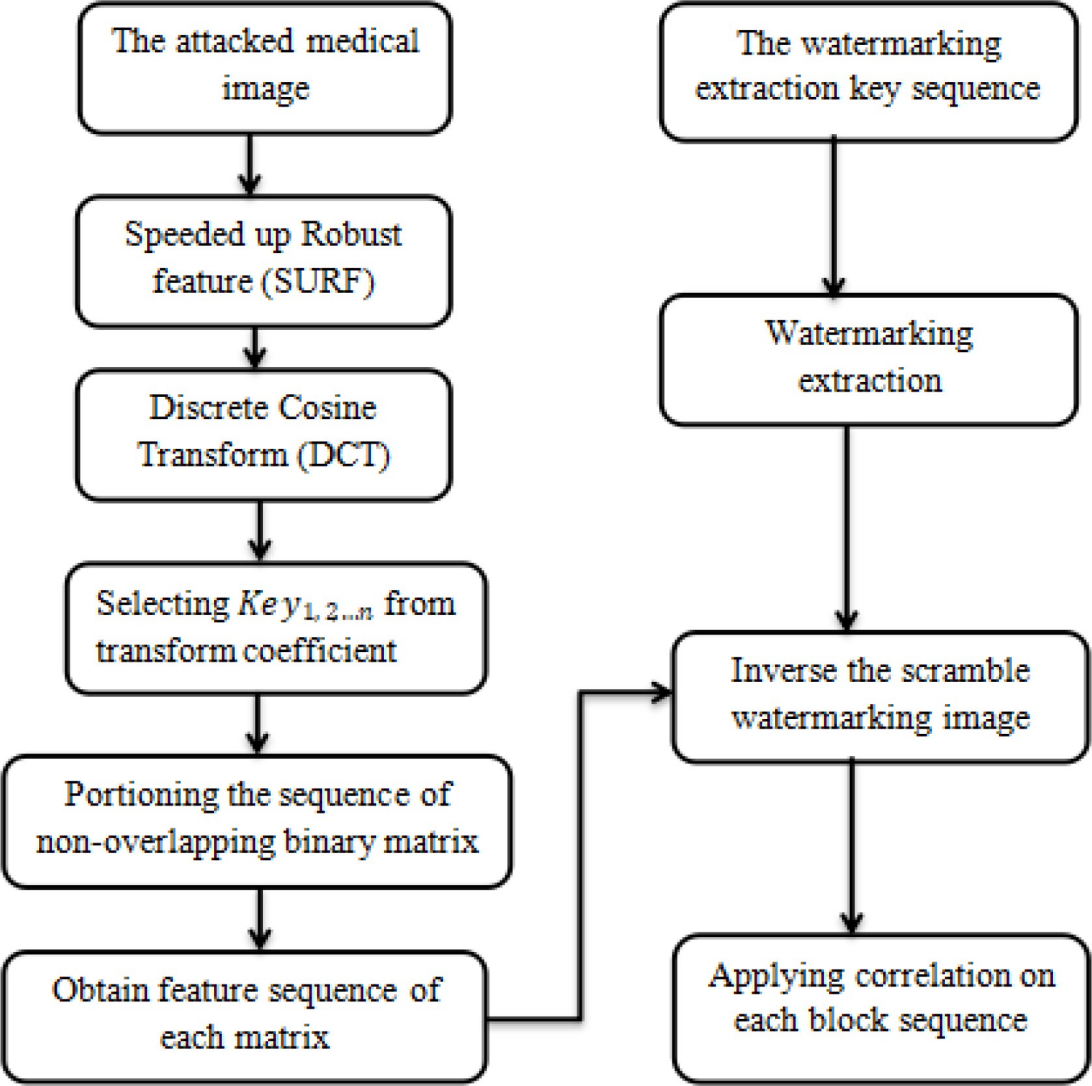
The flow of watermark extraction.

Step 4. Applying the correlation function to check the similarity of the extracted watermark, as shown in Fig 8.

## 5. Experiment

When comparing image stealth watermarking systems, image quality evaluation methods are needed to evaluate the invisibility of digital watermarks. In recent years, the growth of application demand has made research in this area increasingly important. However, humans do not fully understand their visual characteristics. Therefore, no universal image quality evaluation standard has been proposed to date.

The NC (normalized correlation coefficient) and PSNR are used for the image processing validation results. The NC value shows the amount of similarity between the images before and after the watermark. It can be used to evaluate the effectiveness of the proposed algorithm, namely whether the image after the watermark has the same quality as the original image without that watermark. The NC value is calculated, as shown in **Eq** (10). The higher the NC value, the better the watermark similarity, and the stronger the algorithm strength. The second part of the experiment verifies the effect of the algorithm tampering by adding different tampering attacks to different vector images.

**Fig 9:**
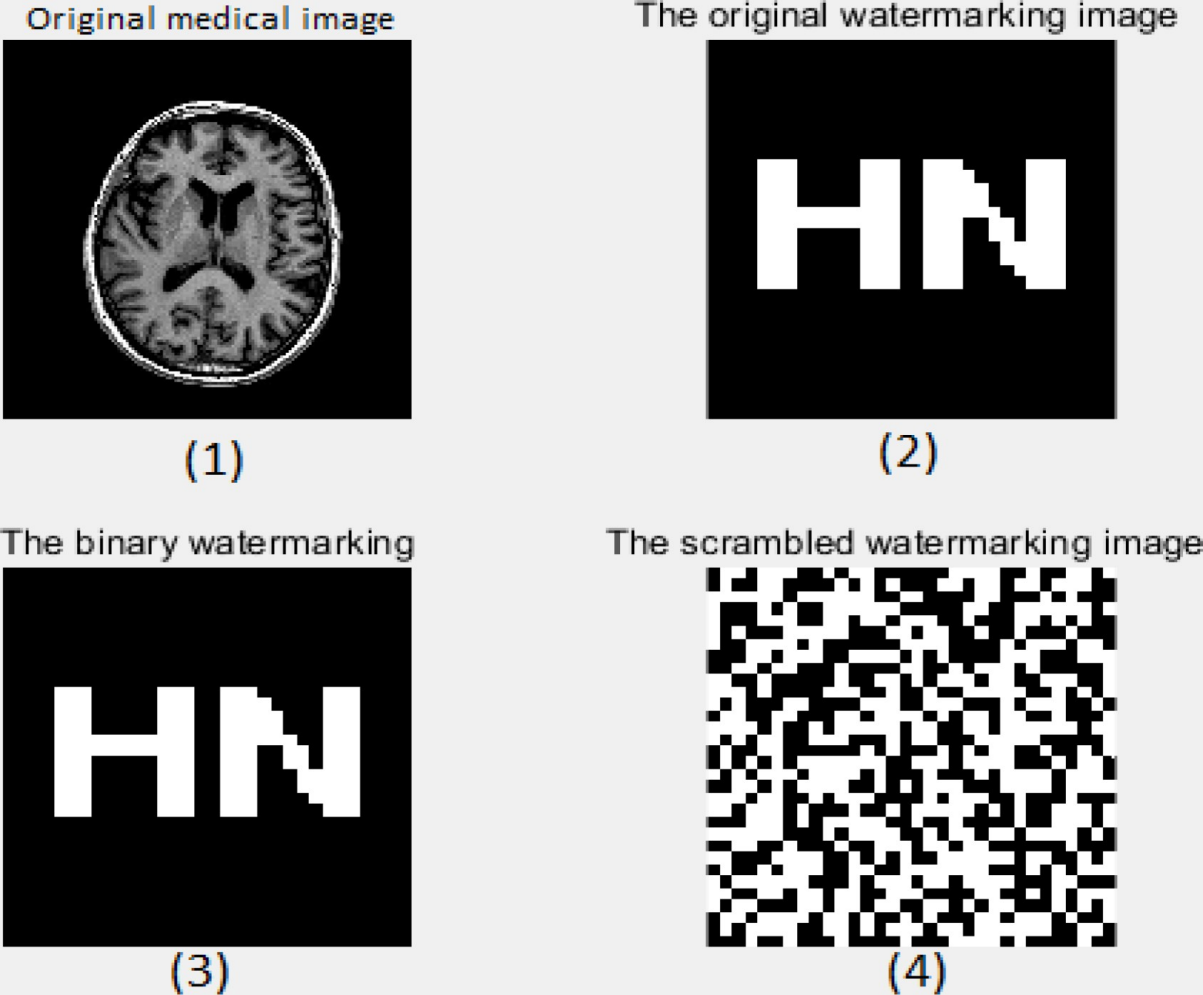
Original image and watermark image.

MATLAB 2018a with an image processing toolbox is used as a simulation platform and medical image with a watermark as well as a binary watermark as in Fig 9(1)–(3), denoted as:

$$W^{k}=\left\{w^{k}\left(i,j\right)|w^{k}\left(i,j\right)=0,1;\;1\leq i,j\leq 32,\;1\leq k\leq 32\right\}$$ (12)

The watermark encrypted by a chaotic map has undergone a great change, and the security is improved. At the same time, the first medical image utilized in the investigation is multi-dimensional imaging of the mind, and the tenth cut image of the therapeutic volume information is chosen as:

F={f(I,j)|f(I,j)∈R;1≤I≤512,1≤j≤512}. (13)

After the calculated watermark recognizes *W^k ' (i*,*j)*, the standardized connection coefficient NC of *W^k (i*, *j)* and *W^k ' (i*, *j)* is determined to decide if there is a watermark attached [30].

The normalized correlation coefficient NC is:

$$NC=\frac{\sum_{i}\sum_{j}W_{\left(i,j\right)}W_{\left(i,j\right)}^{\prime }}{\sum_{i}\sum_{j}W_{\left(i,j\right)}^{2}}$$ (14)

where: *W (i*, *j)* is the originally embedded watermark; *W '(i*, *j)* is the extracted watermark. The larger the NC value, the higher the correlation between the original watermark and the extracted watermark.

The peak signal-to-noise ratio PSNR is used to measure the distortion of a medical image of a water-printed print. The formula is as follows:

$$PSNR=10\;lg\left[\frac{MN\underset{i,j}{max}{\left(I_{\left(i,j\right)}\right)}^{2}}{\sum_{i}\sum_{j}{\left(I_{\left(i,j\right)}-I_{\left(i,j\right)}^{\prime }\right)}^{2}}\right]$$ (15)

where *I_((i*, *j))* and represent the gray value of the pixel of the original image and the watermarked image, respectively, with coordinates *(i*, *j)*; M, N represent the medical image row and column pixel values.

Table 1 shows the low-frequency brain image coefficients after different attacks. After a deep inspection of the coefficient part of the low-frequency transformation, it is observed that values are not changed numerically, and symbols have remained the same. In this research, a low-frequency image is selected for the transformation of symbols and interchanging the data value of 0 with 1 and 1 with 0. This is the SURF DCT coefficient generation technique for low-frequency image sequences, i.e., ‘010111110’. As Table 1 shows, after transformation, the symbol sequences of all attacked images were consistent with the original image, and the NC values were all approximately equal to 1.00.

**Table 1:** SURF-DCT coefficient changes by different attacks for the original image (set of features S1 to S9).

Attacks	PSNR	S1	S2	S3	S4	S5	S6	S7	S8	S9	Feature sequence	NC
Original Image No Attack		−0.442	0.089	−0.248	0.079	0.222	−0.039	0.053	− 0.066	− 3.284	010111110	1
Gaussian noise (4%)	16.08	−1.148	0.113	− 0.095	0.097	−0.089	0.217	− 0.169	−0.098	− 4.916	011111110	0.87
JPEG Compression (15%)	32.03	−0.487	0.079	−0.162	0.185	0.347	− 0.031	0.096	− 0.007	− 0.420	010111110	0.96
Rotation Clockwise (30%)	12.19	−0.455	0.042	−0.189	0.189	0.149	0.013	0.059	0.087	− 3.196	010111110	0.90
Scaling (2)	-	−0.971	0.284	0.036	0.169	0.003	− 0.088	0.088	−0.026	−5.752	011110110	0.83
Translation 30%(right)	10.17	−0.572	0.288	−0.288	0.149	0.084	−0.079	0.164	− 0.093	− 2.774	010111110	0.96
Translation 25% (up)	10.62	−0.376	− 0.046	− 0.153	− 0.068	0.153	−0.017	−0.049	-0.085	− 3.061	010111110	0.94
Cropping 8% (X axis)	-	−0.464	0.121	− 0.279	0.112	0.194	−0.015	0.037	− 0.056	− 3.283	010111110	1

### D. Conventional attacks

Due to the difficulty in performance evaluation between watermarking systems, there is currently no uniform and objective watermark testing standard. It is usually tested by JPEG compression, adding noise, filtering, and shearing. Different conventional attacks were performed on the medical image, and NC estimated values recorded in Table 2.

**Table 2:** Dct and SURF ON conventional attacks.

Conventional attack	Gaussian noise			JPEG Compression		
	2%	4%	8%	10%	15%	20%
PSNR (db)	18.98	16.08	13.32	30.55	32.03	33.48
NC	0.87	0.87	0.83	0.86	0.96	0.86

**Fig 10:**
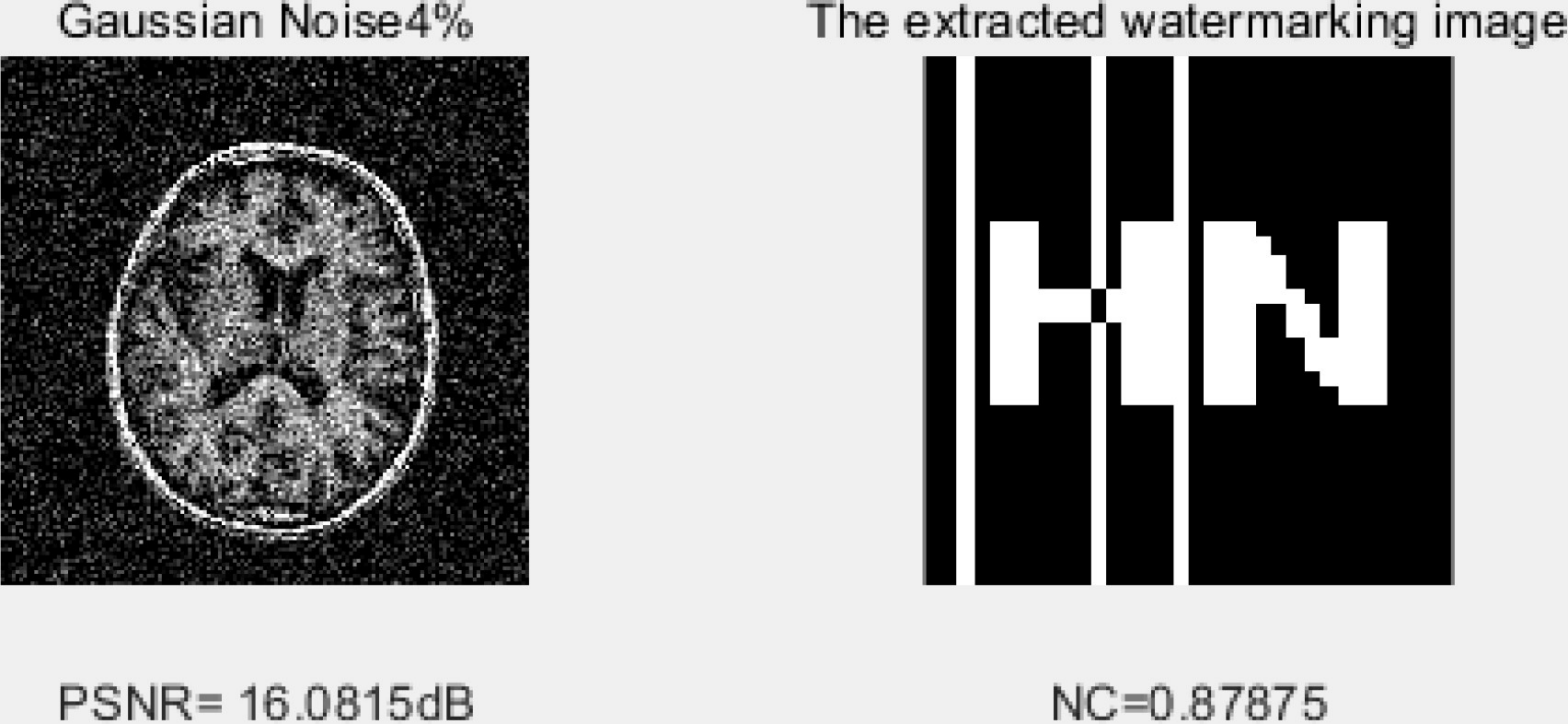
Impact of 4% Gaussian noise.

Gaussian noise. Digital image information suffers from a certain degree of noise attack during transmission and processing. Many digital watermark systems can withstand such attacks within certain limits. Gaussian noise and salt and pepper noise are commonly used noise attack tests. We apply Gaussian noise (4%) attacks to the image, and the estimation of NC is 0.87. The proposed algorithm strongly resists the Gaussian noise attack by providing a higher correlation value of about 87%, as shown in Fig 10.

### E. Geometric attacks

Many watermarking algorithms are relatively fragile for geometric operations and easily removed. Therefore, it is also of interest to study the robustness of digital watermarks after undergoing image geometric distortion transformation. Geometric attacks always have a robustness problem. This algorithm tests a series of Geometric attacks (see Table 3).

**Table 3:** Geometric attacks based on NC and PSNR values using the DCT-SURF.

Geometric Attacks	Strength of attack	PSNR(dB)	NC
Clockwise Rotation	5⁰	15.72	0.86
	20⁰	12.32	0.86
	30⁰	12.19	0.90
Anticlockwise Rotation	4⁰	16.54	0.96
	30⁰	12.19	0.87
	50⁰	11.98	0.92
Scaling	ˣ 0.4	-	0.73
	ˣ 0.8	-	0.82
Translation (Right)	8%	11.63	0.96
	20%	10.63	0.86
	30%	10.17	0.96
Translation (up)	3%	12.83	1
	8%	11.79	0.90
	25%	10.62	0.94
Clipping (Y direction)	8%	-	1
	15%	-	0.90
Clipping (X direction)	8%	-	1
	10%	-	0.94

### 1) Rotation clockwise

**Fig 11:**
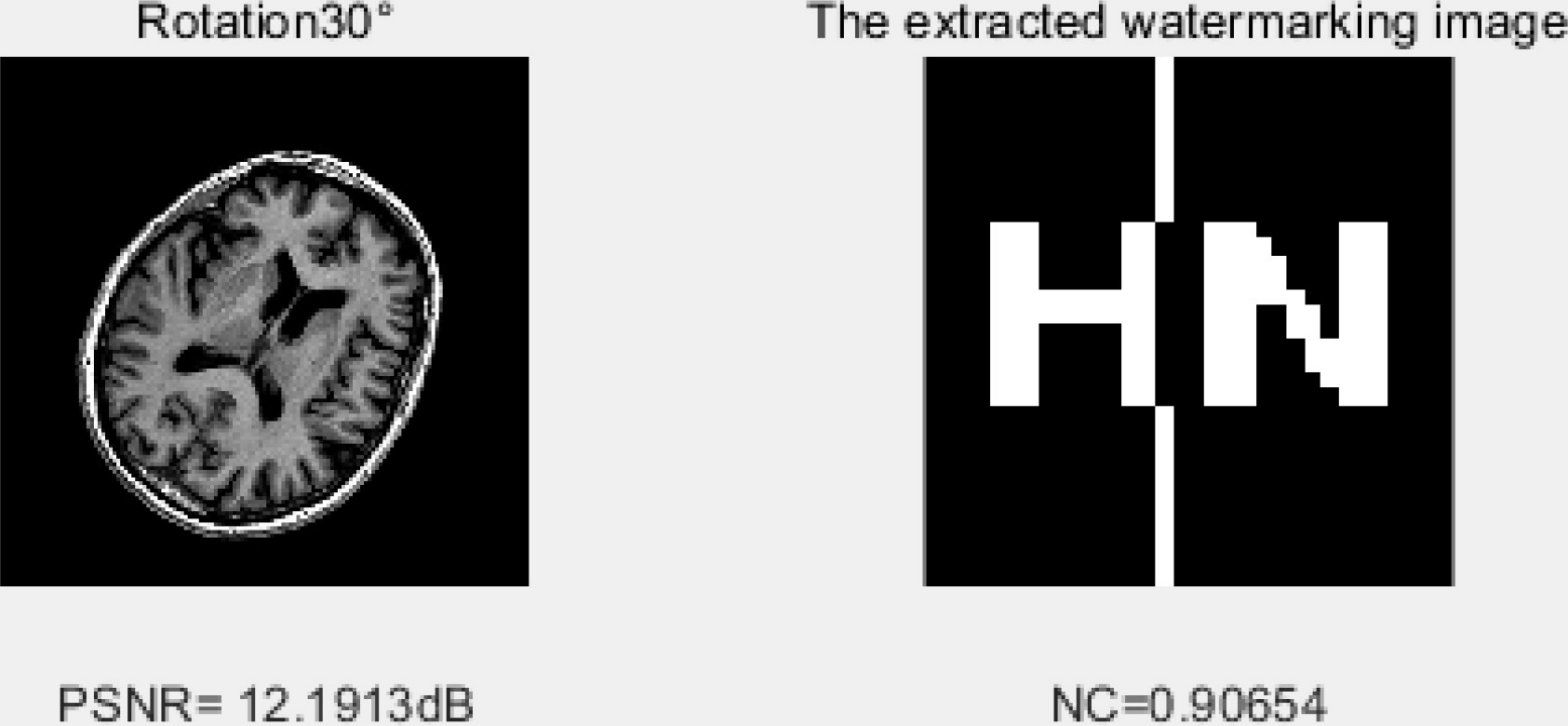
A 30% clockwise rotation after a geometric attack.

Generally, a small angle of rotation does not change the commercial value of the image, but it can redistribute the horizontal features of the image so that the watermark cannot be detected. Fig 11 shows the watermarked image after 30% clockwise rotation of the medical image. The correlation value is 0.90. Our algorithm is robust against the geometric attack by rotation.

### 2) Translation attacks

**Fig 12:**
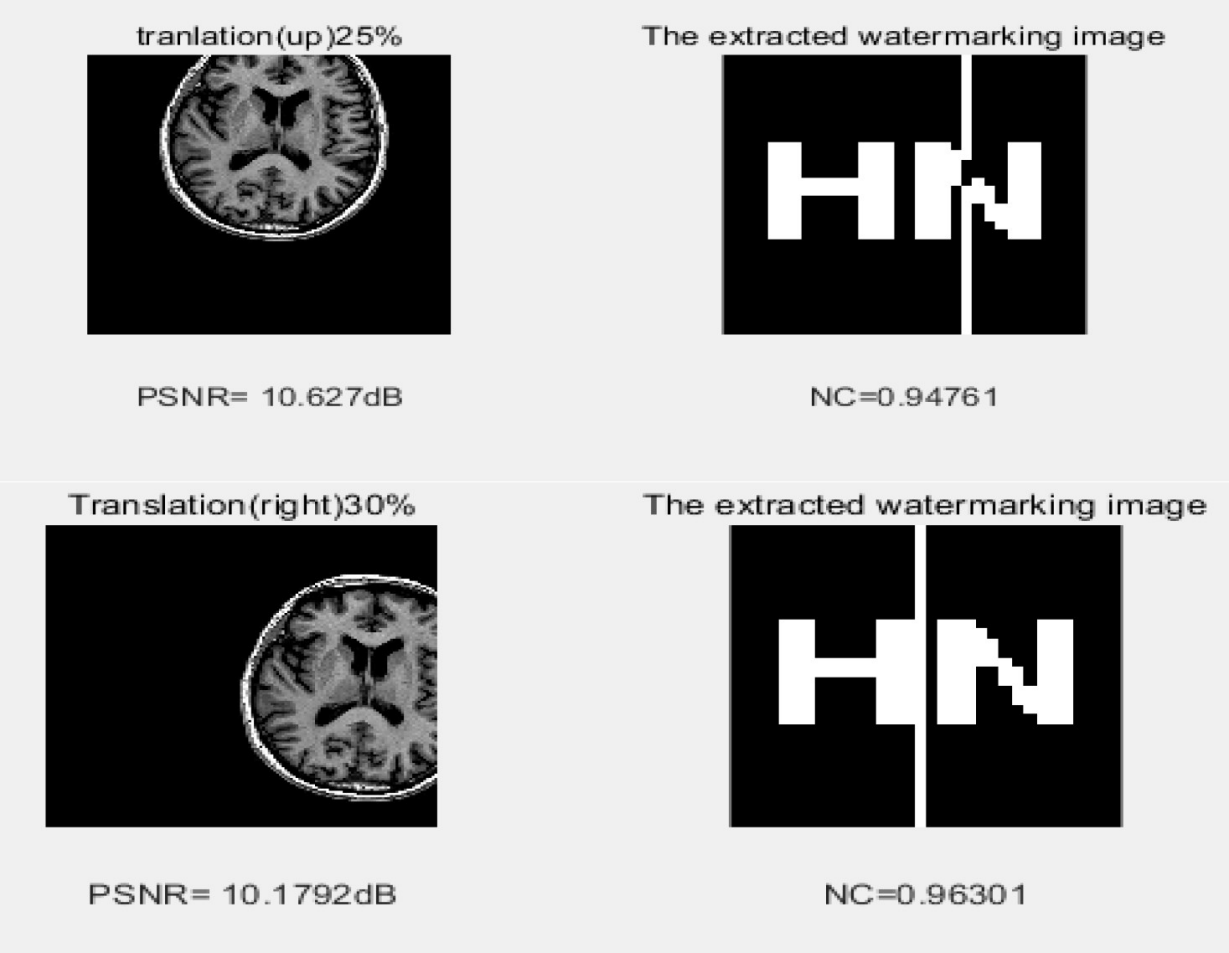
The impact of 25% UP and 30% translation attack on the image.

Different directions and different translation sizes were applied to the image to check the algorithm. The proposed algorithm is robust as results are nearly the same as the watermarked image, indicated by an NC value of .96, as shown in Fig 12.

### 3) Clipping attacks

**Fig 13:**
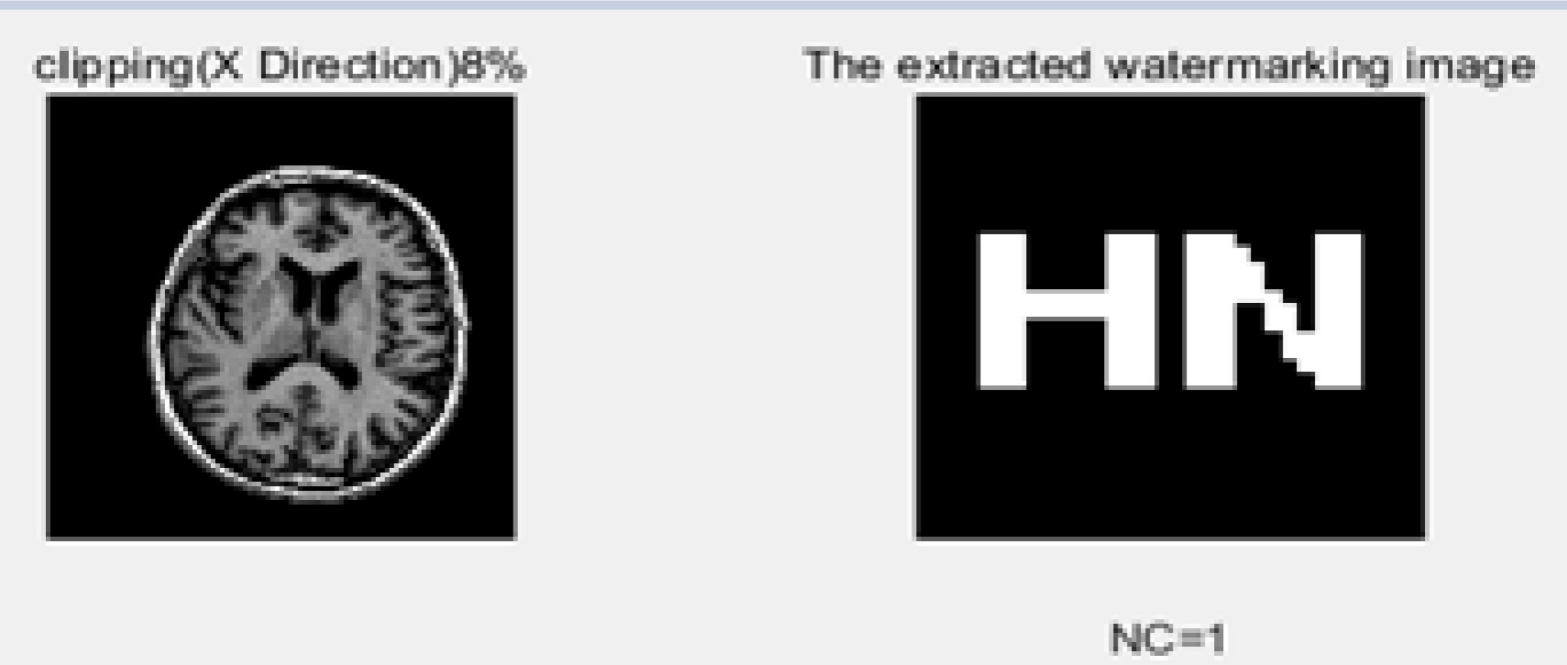
Clipping attack on the x-axis after 8%.

We applied clipping attacks with 8% and 10% strengths on both the x-axis and y-axis. The correlation value results are 100% nearly in both axes. The results after attacks are illustrated in Fig 13. The 100% correlation accuracy indicates that the proposed algorithm performance in clipping attacks is best.

Table 3 provides a detailed comparison of different geometric attacks and shows the efficiency of our proposed algorithm.

#### Performance comparison of algorithms

To prove the effectiveness of our algorithm and improve the quality of results, we compared the algorithm with the other algorithms which were added in our proposed algorithm. Our algorithm has strong robustness to geometric attacks (see Table 4), although the reference SURF [31] and DCT [32] are separately used watermarking algorithms in this paper. From Table 4, it can be clearly seen that a clockwise rotation proposed algorithm is having 90% while SURF performs 75% better than DCT watermarking. However, on a scaling attack, DCT performance is good, and the proposed algorithm correlation is .82, which is better than SURF.

**Table 4:** Proposed algorithm comparison with SURF and DCT.

Attacks strength	NC		
	SURF [31]	DCT[32]	SURF-DCT
Clockwise 30% Rotation	0.75	0.30	0.90
Anticlockwise 30% Rotation	0.72	0.72	0.87
Scaling (×0.8)	0.38	1	0.82
Translation (up) 25%	0.32	0.32	0.94
Cropping (X axis) 8%	1	0.63	1
JPEG (15%) Compression	0.81	1	0.96
Gaussian noise (4%)	0.45	0.96	0.87

Table 5 compares the results of all the algorithms with the proposed algorithm. It is visible that the proposed algorithm performs well under any type of attack. The DWT-DCT-SVD algorithm proposed by Zear et al. has the lowest-performing NC value in each attack. The Amit Kumar Singh et al. algorithm performance is better in scaling attacks using the selective discrete wavelet transformation, thus required a real image for extraction processing. Moreover, the algorithm is embedded in selective parts of the image. However, it is also necessary to perform a wavelet transform with spread spectrum operation, and the embedding of the watermark needs to select different transform layers according to different watermark types [17].

**Table 5:** Comparison of the proposed algorithm with other improved hybrid algorithms.

Attacks	Aditi Zear et al. [33] NC1	Rohit Thanki et al. [34] NC2	Xiaochen yuan et al. [35] NC3	Amit Kumar Singh et al. [36] NC4	Proposed Algorithm NC5 (Medical Image)
Gaussian noise (4%)	0.79	0.76	0.82	0.74	**0.87**
JPEG Compression(15%)	0.38	0.19	0.81	0.56	**0.96**
Rotation Clockwise(30%)	0.23	0.88	0.80	0.79	**0.90**
Scaling (0.8)	0.54	0.61	0.63	**0.99**	0.82
Translation 30%(right)	0.73	0.75	0.69	0.81	**0.96**
Translation 25% (up)	0.56	0.66	0.64	0.73	**0.94**
Cropping 8% (Y axis)	0.79	0.91	0.94	0.85	**1**

Table 6, we compared some latest methods with our proposed method and discussed the features used in every technique. Our method is based on grey medical images and helpful in multiple watermarking as compare to other techniques. Secondly, in QDFT is specially used for color images computation effectively [26] and our algorithm is valid for embedding watermark after encryption using chaotic encryption.

**Table 6:** Features comparison of the proposed algorithm with other techniques.

	Ref [36]	Ref [27]	Ref [26]	Proposed Algorithm
Techniques Used	DWT (Discrete wavelet transformation)+ selective bits	DCT + bit shift using AC	QDFT (Quaternion discrete Fourier Transformation) + extreme pixel adjustment (EPA) + particle swarm optimization (PSO) + mixed modulation (MM)	DCT + SURF + CHAOTIC Encryption
Color or Grey	Color	Color	Color	Grey
Multiple or Single Watermarking	Multiple	Multiple	Single	Multiple

**Fig 14:**
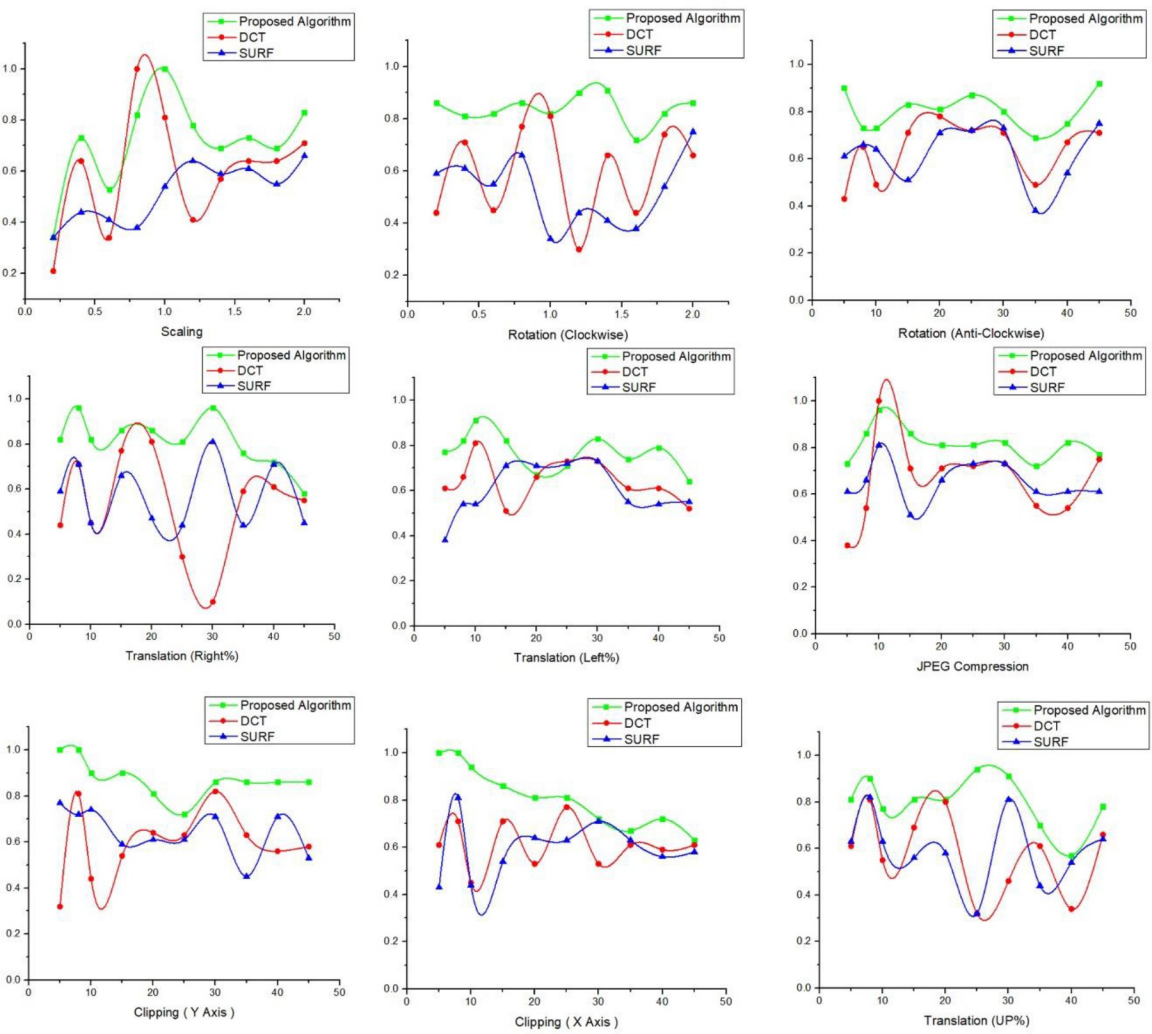
Comparison of proposed algorithm with DCT and SURF on different values of attacks with correlation values.

Conventional and geometric attack tests are performed on the image after embedding the watermark to verify the robustness of the algorithm, and the watermark information is extracted from the image after the attack. DCT and SURF were used on various multiple values to verify the effectiveness of the proposed algorithm on different attacks, as shown in Fig 14. The graph shows the performance of the proposed algorithm correlation values are high compared to DCT and SURF in all attacks on the images. In a few attacks, SURF and the proposed algorithm have some similar values, but overall, the results of the existing methods are not better than our novel algorithm. An algorithm is deemed robust only if the feature matching ratio is high, and the NC value is better. The image similarity before and after the watermark is the best way to measure the robustness.

## 6. Conclusion

Aiming at the problem that the general watermarking algorithm has poor resistance to geometric attacks, this paper uses the invariance of SURF feature points to propose a reversible watermarking algorithm based on SURF feature points to select the DCT region. The approximate sub-band of the watermark is embedded in frequency sub-band of the DCT, and hide watermark. When extracting watermarks, use SURF feature points for geometric correction, and then extract scalable watermarks. The experimental results show that the reversible watermarking technology based on the differential histogram ensures the high quality of the DCT after watermark extraction, SURF correction ensures that the embedded watermark can resist geometric attacks, and combining both guarantees robustness. In the absence of attacks, the undamaged scalable watermark can be extracted. Watermarked images can extract effective watermarks when subjected to signal processing attacks and geometric attacks. They have strong robustness and have certain application value.
